# Impact of the Population Medicine Multimorbidity Intervention in Xishui County (POPMIX) on People at High Risk for Chronic Obstructive Pulmonary Disease Who Experience Mental Health Symptoms: Protocol for the POPMIX-MH Cluster Randomized Controlled Trial

**DOI:** 10.2196/85853

**Published:** 2026-03-06

**Authors:** Yuhao Liu, Wenjin Chen, Shiyu Zhang, Yingping Wang, Zhoutao Zheng, Ke Huang, Xingyao Tang, Zhong Cao, Xunliang Tong, Lei Tang, Jinghan Zhao, Liu He, Lirui Jiao, Tianying Zhao, Yingchi Luo, Qiande Lai, Xiangqin Lyu, Qiushi Chen, Aditi Bunker, Sebastian Vollmer, Pascal Geldsetzer, Dean Jamison, Till Bärnighausen, Ting Yang, Simiao Chen, Chen Wang, POPMIX Group

**Affiliations:** 1 School of Population Medicine and Public Health Chinese Academy of Medical Sciences & Peking Union Medical College Beijing China; 2 School of Health Policy and Management Chinese Academy of Medical Sciences & Peking Union Medical College Beijing China; 3 Center for Disease Control and Prevention of Xishui County Zunyi China; 4 Department of Pulmonary and Critical Care Medicine China-Japan Friendship Hospital Beijing China; 5 National Center for Respiratory Medicine Beijing China; 6 State Key Laboratory of Respiratory Health and Multimorbidity Beijing China; 7 Heidelberg Institute of Global Health Faculty of Medicine and University Hospital Heidelberg University Heidelberg Germany; 8 Department of Pulmonary and Critical Care Medicine Beijing Hospital Beijing China; 9 Institute of Geriatric Medicine Chinese Academy of Medical Sciences Beijing China; 10 Guizhou Medical University Guiyang China; 11 Department of Health Policy and Management Gillings School of Global Public Health University of North Carolina at Chapel Hill Chapel Hill, NC United States; 12 Department of Pulmonary and Critical Care Medicine People’s Hospital of Xishui County Zunyi China; 13 The Harold and Inge Marcus Department of Industrial and Manufacturing Engineering Pennsylvania State University University Park, PA United States; 14 Department of Economics and Centre for Modern Indian Studies University of Göttingen Göttingen Germany; 15 Division of Primary Care and Population Health Department of Medicine Stanford University Stanford, CA United States; 16 Chan Zuckerberg Biohub San Francisco San Francisco, CA United States; 17 Department of Epidemiology and Biostatistics University of California, San Francisco San Francisco, CA United States; 18 Institute for Global Health Sciences University of California, San Francisco San Francisco, CA United States; 19 Department of Global and Population Health Harvard T.H. Chan School of Public Health Harvard University Boston, MA United States; 20 see Acknowledgments

**Keywords:** population medicine, COPD, depression, anxiety, multimorbidity, digital health, China, cluster randomized trial

## Abstract

**Background:**

Chronic obstructive pulmonary disease (COPD) and mental health conditions represent intersecting public health challenges, especially in resource-limited rural China. Existing care models often neglect the psychosocial needs of populations at high risk for COPD, resulting in limited effectiveness of prevention and management strategies. This study evaluates an integrated intervention designed to improve both mental and physical health outcomes among high-COPD-risk individuals with mental health symptoms, using a population medicine framework.

**Objective:**

This study aims to evaluate the effect of an integrated, population medicine–based multimorbidity intervention package among high-COPD-risk individuals with mental health symptoms in Xishui County, Guizhou Province, China.

**Methods:**

We are conducting a 12-month, 2-arm cluster randomized controlled trial across 26 townships in Xishui County, Guizhou, China. A total of 44,000 residents aged ≥35 years were screened using the Chronic Obstructive Pulmonary Disease Screening Questionnaire, identifying 10,000 individuals at high risk of COPD. Among them, 3807 individuals with Warwick-Edinburgh Mental Well-Being Scale scores below 45 were enrolled as participants. Intervention components include digital cognitive behavioral therapy–based mental health support, community screening, chronic disease management, patient education, digital follow-up, and team-based care. The primary outcomes are depressive symptoms (9-item Patient Health Questionnaire), anxiety symptoms (7-item General Anxiety Disorder), and mental well-being (Warwick-Edinburgh Mental Well-Being Scale). Secondary outcomes are control of chronic diseases, physiological and functional indicators such as lung function, health-related quality of life, mental and behavioral health, health care utilization, knowledge of COPD and asthma, productivity loss, and care cascade indicators for chronic conditions.

**Results:**

Data collection for the POPMIX-MH trial began in June 2024. Baseline, 3-month, and 6-month assessments have been completed, and the 12-month follow-up assessments are planned to be completed in March 2026.

**Conclusions:**

This study is the first to integrate psychological support, chronic disease management, and community-based screening into a single scalable intervention package targeting multimorbidity in China. It tests the feasibility of applying population medicine principles, emphasizing integrated, preventive, and population-level care, within primary care systems in low-resource settings. By targeting both mental and physical health, it redefines chronic care beyond traditional organ-specific approaches.

**Trial Registration:**

ClinicalTrials.gov NCT06458218; https://clinicaltrials.gov/ct2/show/NCT06458218

**International Registered Report Identifier (IRRID):**

DERR1-10.2196/85853

## Introduction

### Background

The increasing complexity of chronic disease epidemiology, characterized by the convergence of biomedical, behavioral, psychological, and structural risk factors, has challenged the effectiveness of conventional, disease-centered care models [1,2]. Population medicine has therefore been proposed as an approach that emphasizes preventive, integrated, population-level care to realign health systems with the growing burden of multimorbidity and health inequalities [3]. The Lancet Commission’s “50-by-50” goal highlights tobacco-related noncommunicable diseases (NCDs) as a priority area for action and estimates that they account for approximately 1 year of the 3.5-year life expectancy gap between China and the North Atlantic regions [4,5]. These patterns underscore the need to address tobacco-related NCDs at the population level through early identification and comprehensive disease management.

Chronic obstructive pulmonary disease (COPD) is the fourth leading cause of death and disability-adjusted life years globally and is projected to affect nearly 600 million people by 2050, with a disproportionate rise among women and populations in low- and middle-income countries [6,7]. In China, the disease accounted for over 11 million deaths in 2021 and continues to impose a substantial economic burden, with estimated costs exceeding Int $1.3 trillion between 2020 and 2050 [6,8]. Despite its high burden, COPD remains severely underdiagnosed and undertreated, particularly in primary care and low-resource settings [9]. Recent research has revealed substantial gaps across the COPD care cascade in China, particularly in early detection, formal diagnosis, treatment initiation, and symptom control. Data from the “Happy Breathing Programme,” a large-scale population-based COPD screening and management initiative in China, reveal major gaps along the care cascade: among 29,201 patients with COPD, 41.0% had previously undergone spirometry testing, 17.6% had been formally diagnosed, and 8.5% were currently receiving treatment. Among those receiving treatment, 4.6% had mild or no exacerbations in the prior year, and 3.9% had experienced no exacerbations [10]. The severity of this care cascade breakdown underscores the urgent need for proactive, community-level interventions aimed at identifying and supporting high-risk individuals before irreversible disease progression occurs.

COPD rarely occurs in isolation but typically forms part of complex multimorbidity profiles that worsen quality of life and clinical outcomes [11]. Among the various comorbid conditions, mental health disorders, particularly depression and anxiety, are highly prevalent yet often overlooked [11]. Meta-analytic and large cohort evidence suggests that the prevalence of depression in people with COPD is around 25%-30% compared with about 10% in the general adult population [12], corresponding to roughly a 2- to 3-fold higher risk of depression and anxiety than in the general population [13,14]. A national multicenter study found that patients with COPD with comorbid depression had a markedly greater symptom burden and poorer health-related quality of life, yet only 39% were receiving antidepressant treatment and 22% had access to mental health counseling [15].

Current COPD prevention and management frameworks, in both clinical guidelines and public health programs, still focus mainly on biomedical risk factors such as lung function decline and tobacco use, while mental health receives little attention, particularly among high-risk individuals who do not yet meet diagnostic criteria and are therefore rarely screened or systematically assessed for psychological distress [16-20]. Existing evidence on COPD largely comes from diagnosed patients, among whom depression and anxiety are highly prevalent yet frequently underrecognized and undertreated, suggesting that similar or greater unmet mental health needs may exist in undiagnosed high-risk populations who remain outside the formal care system [21,22]. At the same time, most prevention efforts for COPD continue to target physiological indicators, and only a few models address multimorbidity and psychosocial needs in an integrated manner, even though integrated disease management that combines respiratory care, education, and mental health support has been shown to improve self-management, reduce exacerbations, and enhance quality of life [18,19,23-25]. Cognitive behavioral therapy (CBT) is an effective and scalable intervention for anxiety and depression that can be delivered digitally via smartphones, yet such integrated, technology-enabled approaches have rarely been applied to individuals at high risk for COPD; our multicomponent intervention, which incorporates digital CBT, spirometry-based risk stratification, community education, and behavioral self-management support, is designed to address this gap in resource-limited settings [23,25-29].

### Objectives

This research forms part of the Population Medicine Multimorbidity Intervention in Xishui County (POPMIX) project, which focuses on creating and assessing evidence-informed, multicomponent approaches for managing chronic conditions and their multimorbidity in resource-limited settings. This trial targets individuals at high risk of COPD who also exhibit mental health symptoms. We will examine whether a population-based, integrated intervention for multiple long-term conditions affects the primary outcomes—symptoms of depression, symptoms of anxiety, and overall mental well-being. Additionally, we will evaluate the intervention’s impact on several secondary outcomes, including the number of controlled multimorbid chronic conditions; forced expiratory volume in 1 second (FEV_1_); quality-of-life measures; patient knowledge of COPD and asthma; indicators of COPD symptoms; levels of smoking dependence; care cascade indicators for COPD, asthma, hypertension, and type 2 diabetes; patterns of health care services utilization; indicators of health risk behaviors; and productivity loss.

## Methods

### Trial Design

**Figure 1:**
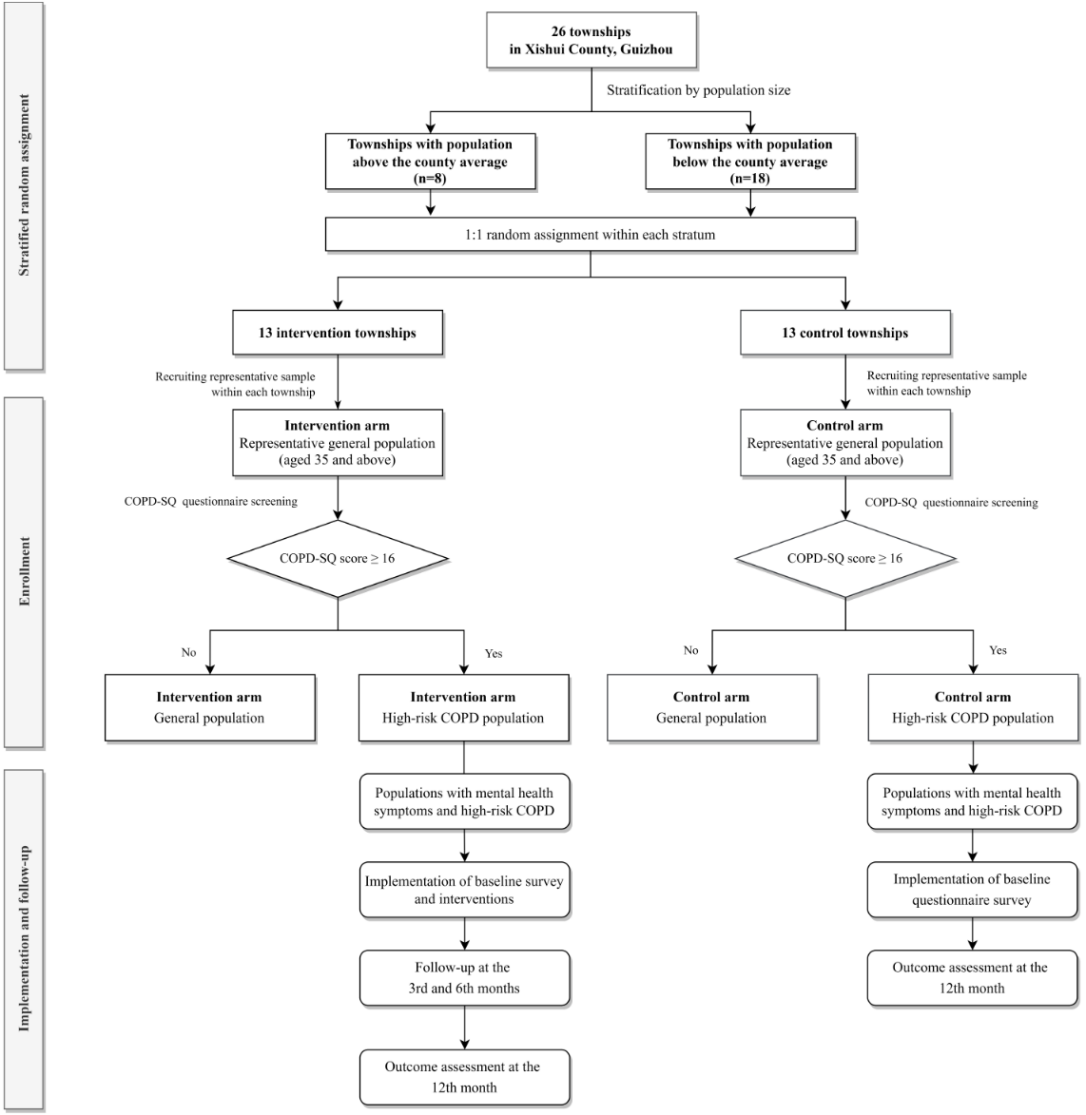
Trial flowchart. COPD: chronic obstructive pulmonary disease; COPD-SQ: COPD Screening Questionnaire. See Multimedia Appendix 4 for a higher resolution version of this figure.

This investigation employed a parallel, 2-arm, stratified cluster randomized controlled trial (cRCT) design, implemented in Xishui, Guizhou, China (Figure 1). The study protocol was developed in strict accordance with the guidance of the Standard Protocol Items: Recommendations for Interventional Trials (SPIRIT) 2025 Statement [30]. Compliance with SPIRIT 2025 is detailed in Multimedia Appendix 1, which includes the completed checklist for this protocol.

Townships were stratified according to whether they were above or below the county average population size. Within each stratum, townships were randomly assigned (1:1) to the intervention or control arm using computer-generated randomization. Participants were recruited from a government-provided roster of permanent residents aged 35 years or older as of May 10, 2024, with individuals enrolled from each township.

The COPD Screening Questionnaire (COPD-SQ) was used to identify individuals at high risk of COPD. Developed by Zhou et al [31], this instrument comprises 7 items: age, cumulative smoking history, BMI, cough, breathlessness, family history of respiratory diseases, and exposure to cooking-related smoke. It generates a total score ranging from 0 to 38, with higher scores indicating greater COPD risk. Validated through multiple epidemiological surveys and community health screenings, the COPD-SQ is recommended for primary care–based COPD screening in China. For this study, participants scoring 16 or higher on the COPD-SQ were considered eligible for inclusion, thereby ensuring that the cohort comprised individuals identified as high risk for COPD.

### Setting

This study was implemented across 26 township-level clusters in Xishui County, a mountainous area in northern Guizhou Province, China. A rural county designated as China’s National Comprehensive Primary Health Experimental Area, Xishui presents a unique juxtaposition of policy-driven innovation and socioeconomic challenges. With a gross domestic product per capita (48%) below the national average in 2024 and limited human capital development, the county exemplifies a resource-constrained rural setting. Notably, Guizhou Province also has one of the highest prevalences of smoking in China (at 37.9%), driving a high regional burden of tobacco-related NCDs such as COPD [32]. These conditions, combined with the county’s experimental role in health system transformation, have established Xishui as an ideal setting in which to evaluate scalable multimorbidity interventions tailored to underserved populations.

### Trial Participants (Inclusion and Exclusion Criteria)

Participants were eligible for inclusion if they were aged 35 years or older and met the study definition of a high-COPD-risk individual with mental health symptoms. Such individuals were defined as having a COPD-SQ score of 16 or higher, indicating elevated risk for COPD, and a Warwick-Edinburgh Mental Well-Being Scale (WEMWBS) score of 45 or lower, reflecting reduced mental well-being. To ensure stability and adherence to the intervention, only participants who had lived in the same township for at least 3 months before enrollment and who planned to remain in the township for the subsequent 12 months were included. This residency requirement helped limit population mobility and reduce potential dilution or contamination of intervention effects.

Individuals exhibiting severe cognitive disorders that substantially compromised comprehension, decision-making capacity, or intervention adherence were excluded. Likewise, participants lacking full independence in performing activities of daily living were ineligible. These criteria ensured that enrolled individuals could actively participate in the intervention and follow-up procedures while reducing potential confounding factors.

### Intervention

Participants in the intervention group were provided with access to a multicomponent intervention package. This package integrates key strategies, including community-based risk screening for COPD and mental health problems, digital CBT delivered via the EmoEase program, risk-stratified management of chronic respiratory and cardiometabolic conditions, smoking cessation support and health education, and a pay-for-population incentive mechanism for primary care providers within routine primary care (Multimedia Appendix 2). The intervention package was designed in collaboration with policy makers, aligning with the specific needs and challenges of Xishui County. Policy makers primarily focused on the overall effectiveness of the intervention package rather than on individual components. Accordingly, the integrated design of the package is highly relevant to the local context, aiming to address broad community health goals rather than isolating the effects of each subintervention.

**Figure 2:**
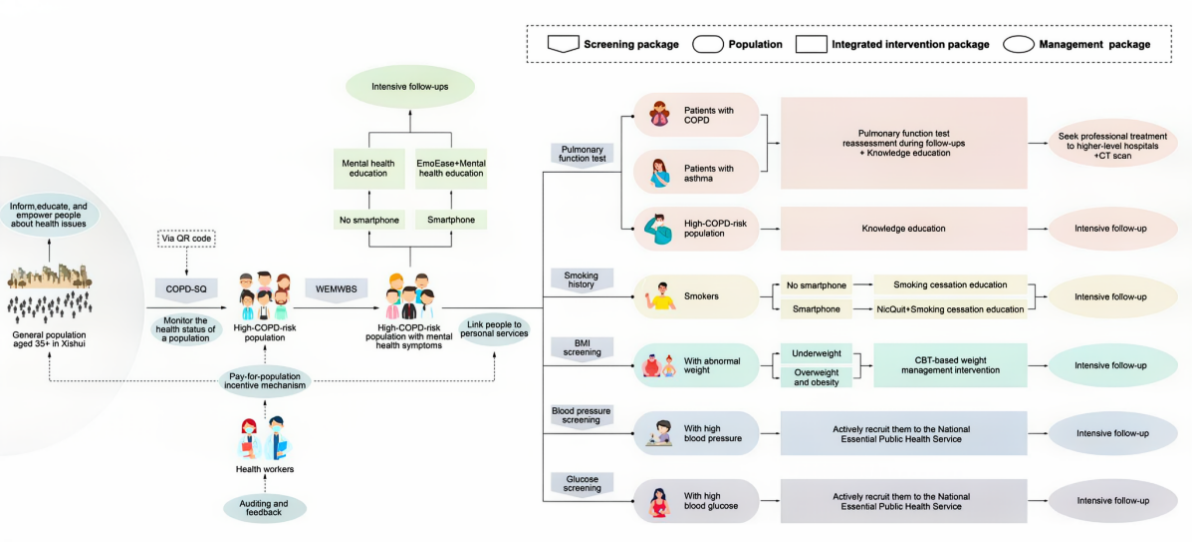
Integrated pathway of the multicomponent intervention package. CBT: cognitive behavioral therapy; COPD: chronic obstructive pulmonary disease; COPD-SQ: COPD Screening Questionnaire; CT: computed tomography; WEMWBS: Warwick-Edinburgh Mental Well-Being Scale. See Multimedia Appendix 4 for a higher resolution version of this figure.

Developed using iterative prototyping and stakeholder input, the strategies within this package are all established as cost-effective and are specifically promoted by the Lancet Commission on Investing in Health [4]. Table 1 summarizes the distinct eligibility criteria applicable to each intervention. The control group receives usual care only. In Xishui, usual care largely follows a traditional, reactive model, in which residents seek care on their own initiative when they experience symptoms rather than through proactive population management. As a national pilot county for comprehensive primary health care reform, Xishui has relatively favorable reimbursement policies for chronic diseases. A visualized schematic flow of the integrated intervention logic is presented in Figure 2. This figure maps the progression from initial risk screening through intervention stratification and follow-up for multiple chronic conditions. The illustration also demonstrates how the EmoEase program and chronic disease pathways are embedded within a broader population medicine framework.

**Table 1:** Eligibility criteria for interventions.

Population level and target population		Intervention	Eligibility criteria
**General population**			
	General population	Health education	N/A^a^
	General population	Online screening with the COPD^b^ Screening Questionnaire, followed by WEMWBS screening for identified high-COPD-risk individuals	Permanent residents aged 35 years and above
**Patients with COPD or high-COPD-risk population**			
	High-COPD-risk population	Community-based spirometry pulmonary function tests, interpretation of results, and health education	Individuals with COPD-SQ^c^ score ≥16
	High-COPD-risk population with mental health symptoms	Digital mental health interventions and health education	Individuals with WEMWBS^d^ score <45 who own a smartphone
	High-COPD-risk population with mental health symptoms	Health education for mental health symptoms	Individuals with WEMWBS score <45
	Smokers within the high-COPD-risk population with mental health symptoms	Digital interventions for smoking cessation and mental health, along with health education	Currently smoking or have quit within the last 6 months and own a smartphone
	Patients with COPD or high-COPD-risk population with mental health symptoms who fail to conduct pulmonary function tests	Encouragement to conduct a computed tomography scan and seek professional medical treatment	Individuals with FEV_1_^e^/FVC^f^ <0.7 after bronchodilation, or high-COPD-risk population who fail to conduct a pulmonary function test for any reason
	High-COPD-risk population with mental health symptoms, and hypertension	Hypertension management and education	Systolic blood pressure ≥140 mmHg or diastolic blood pressure ≥90 mmHg
	High-COPD-risk population with mental health symptoms, and type 2 diabetes mellitus	Diabetes management and education	Fasting blood glucose ≥7.0 mmol/L or random blood glucose ≥11.1 mmol/L
	High-COPD-risk population with mental health symptoms, and abnormal weight	Weight abnormality interventions	Individuals with a BMI < 18.5 kg/m^2^or a BMI ≥ 24.0 kg/m^2^
	Smokers within the high-COPD-risk population with mental health symptoms	Health education for smokers to support smoking cessation	Currently smoking or have quit within the last 6 months
**Health providers**			
	Health providers	Intrinsic incentive mechanism	Primary care providers who engage with the investigation and intervention
	Health providers	Extrinsic incentive mechanism	Primary care providers who engage with the investigation and intervention

^a^N/A: not applicable.

^b^COPD: chronic obstructive pulmonary disease.

^c^COPD-SQ: COPD Screening Questionnaire.

^d^WEMWBS: Warwick-Edinburgh Mental Well-Being Scale.

^e^FEV_1_: forced expiratory volume in 1 second.

^f^FVC: forced vital capacity.

**Figure 3:**
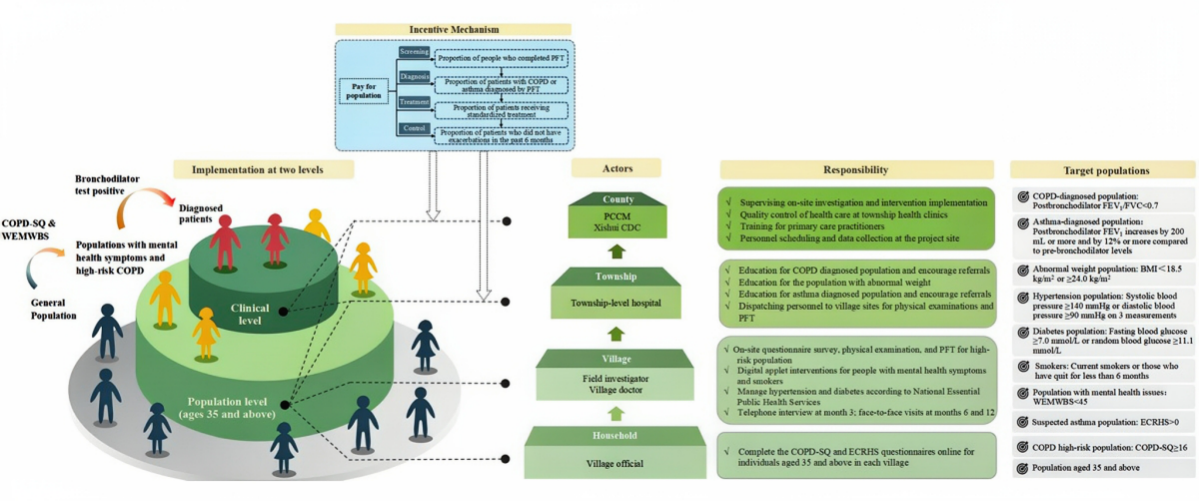
Implementation structure and execution mechanism of the multicomponent intervention package. CDC: Centers for Disease Control and Prevention; COPD: chronic obstructive pulmonary disease; COPD-SQ: COPD Screening Questionnaire; ECRHS: European Community Respiratory Health Survey; FEV1: forced expiratory volume in 1 second; FVC: forced vital capacity; PCCM: pulmonary and critical care medicine; PFT: pulmonary function test; WEMWBS: Warwick-Edinburgh Mental Well-Being Scale. See Multimedia Appendix 4 for a higher resolution version of this figure.

In addition to the intervention components and care pathways described in Figure 2, implementation of the multicomponent intervention package was supported by a multilevel delivery structure and a performance-linked incentive mechanism. Figure 3 illustrates the organizational structure, population stratification, incentive design, and distribution of responsibilities across administrative levels (county, township, village, and household) for delivering the intervention in Xishui. This figure also specifies the eligibility criteria for each target subpopulation, clarifying how risk stratification and service delivery were operationalized in the field.

### Specific Interventions

#### Overview

Specific interventions as part of the multicomponent intervention package are described in the following sections.

#### Health Education

Health education is provided universally to all permanent residents living in townships randomized to the intervention arm. The content includes basic information about COPD; mental health; and common risk factors such as tobacco exposure, poor diet, and lack of physical activity. Educational materials are distributed in both printed and digital formats, such as videos/messages distributed via WeChat (Tencent Holdings Limited). Health workers conduct door-to-door education sessions and use public broadcasts and posters in villages to raise awareness and promote behavior change. These activities aim to improve population-level health literacy and early symptom recognition.

#### Psychological Health Questionnaire Screening

To facilitate the early detection of psychological distress, we implement mental health questionnaire screening at baseline. All high-COPD-risk populations aged 35 years and above are invited to complete a brief mental health assessment in the baseline questionnaire, which consists of the WEMWBS to measure overall mental well-being, the 9-item Patient Health Questionnaire (PHQ-9) to assess depressive symptoms, and the 7-item Generalized Anxiety Disorder (GAD-7) scale to assess anxiety symptoms. Screening results are used to identify individuals who may benefit from subsequent digital mental health interventions and tailored follow-up support.

#### Online Screening for COPD

To facilitate the early identification of the high-COPD-risk individuals, the study implements an online screening mechanism using digital tools. A QR code linking to the COPD-SQ is distributed to all eligible representative permanent residents aged 35 years and above. Community health workers assist participants in completing the online forms when needed. This approach enables broad population coverage, immediate risk assessment, and real-time referral for spirometry testing and education. Screening results are stored in the digital health system to support follow-up planning.

#### Community-Based Spirometry Pulmonary Function Testing and Health Education

High-COPD-risk individuals in the intervention group receive real-time pop-up alerts directing them to a designated community gathering place for spirometry testing, which is conducted using the BH-AX-MAPG spirometry equipment (BreathHome). Prebronchodilator spirometry is conducted at baseline and 12 months for the high-COPD-risk population; postbronchodilator spirometry is conducted at baseline and 12 months for patients with COPD; and prebronchodilator spirometry is conducted at 6 months for high-COPD-risk participants with prebronchodilator FEV/FVC<70% at baseline. Individuals who screen positive for COPD at baseline are referred to the county hospital for computed tomography and a definitive diagnosis. In addition, they receive health education on the risks of COPD and strategies for its prevention and management. This education is delivered verbally by primary health care providers and supplemented with printed materials distributed by trained community health workers or general practitioners.

#### Mental Health Digital Health Interventions

A CBT-based digital mental health intervention, EmoEase, a WeChat (Tencent Holdings Limited)-based application, is offered to individuals experiencing mental health symptoms (WEMWBS score <45) who also have a smartphone [33]. The intervention includes structured psychoeducational content, mood tracking, and guided CBT exercises designed to enhance emotional regulation and reduce symptoms of anxiety and depression [33]. The content is tailored to highlight the interplay between mental health and respiratory symptoms, aiming to improve coping strategies, promote treatment adherence, and support sustainable behavior change. The program is self-paced and optimized for accessibility in low-resource settings, with additional support provided by primary health care providers to encourage ongoing engagement.

#### Health Education for High-COPD-Risk Individuals With Mental Health Symptoms

Health education is offered to high-COPD-risk individuals with co-occurring mental health symptoms, defined by a WEMWBS score below 45. This health education includes guidance on managing mental health symptoms and is delivered verbally by trained community health workers or general practitioners, supplemented with printed materials for distribution.

#### Smoking Cessation Digital Health Interventions

NicQuit is a WeChat-based digital smoking cessation intervention that includes CBT modules focused on smoking cessation strategies, methods for coping with triggers, and reinforcement techniques to maintain abstinence. It is designed for smokers who are currently smoking or who have quit within the last 6 months. The intervention targets individuals familiar with smartphone technology, ensuring accessibility and usability. Personalized notifications and reminders are delivered through the WeChat platform to encourage regular engagement with the cessation plan and maintain adherence.

#### Encouragement to Conduct Computed Tomography Scan and Seek Professional Medical Treatment

Participants diagnosed with COPD or asthma through spirometry—defined as those with a postbronchodilator FEV_1_-to-forced vital capacity (FVC) ratio of <0.7 for COPD and those with a ≥200 mL and ≥12% improvement in FEV_1_ postbronchodilation for asthma—are encouraged to seek professional medical treatment at higher-level hospitals for further diagnosis and management. High-risk participants who do not undergo pulmonary function testing for any reason are encouraged to receive a computed tomography scan and seek formal diagnosis and medical treatment at a higher-level hospital.

#### Hypertension and Diabetes Management

The goal of this intervention is to actively enroll high-COPD-risk individuals with mental health symptoms and blood pressure higher than 140/90 mmHg or random blood glucose higher than 11.1 mmol/L (or fasting blood glucose ≥7.0 mmol/L) into the National Essential Public Health Service in China. These participants are also provided with health education on abnormal blood pressure or blood glucose [34,35].

#### Weight Abnormality Interventions

Individuals with a BMI <18.5 kg/m^2^ (underweight) or a BMI ≥24.0 kg/m^2^ (overweight and obesity) are considered to have weight abnormalities. CBT-based motivational interviewing is used to guide participants in self-identifying weight-related barriers. During the intervention, participants are asked CBT-informed questions designed to encourage active reflection on the inconveniences and conveniences of being underweight or overweight.

#### Health Education for Smokers to Support Smoking Cessation

Smokers in the intervention group receive targeted health education to reinforce the importance of smoking cessation. This education focuses on the health risks associated with smoking and the benefits of quitting, providing evidence-based information and practical advice. The health education is delivered verbally by primary health care providers and supplemented with printed posters, online videos/messages for distribution.

#### Pay-for-Population Mechanism

A novel pay-for-population mechanism has been introduced to actively engage primary care providers in the intervention by aligning their compensation with population health goals. This approach ties provider payment to 4 key stages of care: screening, diagnosis, treatment, and control. Health providers in township hospitals are rewarded based on the proportion of residents aged 35 years and above who complete pulmonary function testing; the proportion of individuals diagnosed with COPD among those identified as high risk in the initial screening (defined as COPD-SQ score >16); the proportion of patients with confirmed COPD receiving standardized inhaled treatment relative to all confirmed cases; and the proportion of patients with confirmed COPD who have not experienced acute exacerbations in the past 6 months. The county hospital’s respiratory department and the county Center for Disease Control and Prevention are assessed using the same 4 indicators, with data aggregated across all 13 intervention townships. In addition to offering providers extrinsic, results-based financial incentives, the program provides specialized training and capacity-building opportunities to appeal to intrinsic motivation, support proactive service delivery, and foster a strong sense of responsibility for population health (see Multimedia Appendix 2 for details).

Importantly, the POPMIX-MH intervention does not increase the number of primary care providers or extend their contracted working hours. All activities are delivered by the existing workforce within routine service schedules, with capacity enhanced through focused training on COPD risk assessment, mental health screening, and population-based follow-up. These activities have not reduced access to routine care for nonparticipants; on the contrary, opportunistic screening has enabled providers to identify additional residents with previously unmet health needs and to offer appropriate counseling or referral when indicated.

To enhance clarity, Table 2 summarizes all intervention and control activities at baseline, during the 3- and 6-month follow-ups, and at the 12-month final assessment.

**Table 2:** Components and timeline of the intervention and control conditions.

Time point	Intervention group	Control group
Baseline	Online spirometry questionnaire screening Psychological health questionnaire screening Collection of demographic and health information Distribution of the mental health intervention and other chronic obstructive pulmonary disease–related interventions (see Figure 1, Table 1 for details)	Online spirometry questionnaire screening Psychological health questionnaire screening Collection of demographic and health information
Month 3	Follow-up assessment of intervention adherence and psychological health status; delivery of booster sessions	N/A^a^
Month 6	Follow-up assessment of intervention adherence and psychological health status; delivery of booster sessions	N/A
Month 12	Final outcome assessment	Final outcome assessment

^a^N/A: not applicable.

### Outcomes

We appraise primary and secondary outcomes among high-COPD-risk individuals with concurrent psychological distress symptoms. Following intention-to-treat allocation to the intervention or control arm, participants are tracked longitudinally for 12 months. Outcome and covariate data are collected at 3 months (via telephone for the intervention group only), at 6 and 12 months (via in-person visits) after enrollment. Specific secondary end points (chronic disease knowledge, health service use, and count of controlled chronic conditions) are measured solely at 12 months. Table 3 presents the definitions and measurement procedures for the primary outcomes; secondary outcomes are listed in Table S1 in Multimedia Appendix 3.

**Table 3:** Primary outcomes of the cluster randomized controlled trial.

Primary outcomes	Description
Depression symptoms	Definition: Emotional disorders, including sadness, loss, and anger Functional form: Continuous Measurement: Score on the 9-item Patient Health Questionnaire items [36], which ranges from 0 to 27, with higher scores representing more severe depression symptoms
Anxiety symptoms	Definition: Unpleasant state of inner turmoil Functional form: Continuous Measurement: Score on the 7-item General Anxiety Disorder scale [37], which ranges from 0 to 21, with higher scores representing more severe anxiety symptoms
Warwick-Edinburgh Mental Well-Being Scale	Definition: A score reflecting the overall mental health state Functional form: Continuous Measurement: Score on the Warwick-Edinburgh Mental Well-Being Scale [38], which ranges from 14 to 70, with lower scores representing worse general mental health

### Timeline

Trial initiation occurred on June 17, 2024, with recruitment spanning townships in Xishui County. Participants in the intervention group will complete structured assessments at 4 time points: baseline (in person), 3-month follow-up (telephone), 6-month follow-up (in person), and 12-month follow-up (in person). Postconsent baseline assessments include comprehensive physical examinations and pulmonary function tests. Pulmonary function test protocols are stratified: prebronchodilator spirometry is conducted at baseline and 12 months for the high-COPD-risk population; postbronchodilator spirometry is conducted at baseline and 12 months for patients with COPD; and prebronchodilator spirometry is conducted at 6 months for high-COPD-risk participants with prebronchodilator FEV/FVC<70% at baseline. All baseline procedures are supervised by trained site personnel to ensure protocol adherence. Subsequent follow-ups include a telephone-based 3-month assessment (tracking intervention compliance and health status) and in-person 6- and 12-month visits replicating baseline protocols. Control group activities include baseline questionnaires, and 12-month on-site surveys. The prospective cohort timeline and data collection schedule are detailed in Table S2 in Multimedia Appendix 3.

### Sample Size

The study encompassed all 26 townships in Xishui County, which were allocated 1:1 to the intervention or control group through stratified randomization. We enrolled a population-representative sample of 44,000 individuals aged 35 years or older. COPD risk screening was conducted universally using the COPD-SQ, administered digitally via QR code. Based on an anticipated 10% attrition rate and an estimated high-COPD-risk prevalence of 25% in this age group, we projected 10,000 high-risk participants.

Three primary outcomes were prespecified: overall mental well-being measured by the WEMWBS, depressive symptoms measured by the PHQ-9, and anxiety symptoms measured by the GAD-7. The sample size calculation was based on the WEMWBS, which served as the primary well-being end point. To control for multiple comparisons across the 3 primary outcomes, a Bonferroni correction was applied by dividing the significance threshold by 3. The parameters used for the WEMWBS-based sample size calculation, including an SD of 10.59 and an anticipated mean difference of 5.95, were conservatively derived from a prior longitudinal observational study of online psychiatric assessments [39]. Accounting for a 2-sided α of 5%, 80% power, an intraclass correlation coefficient (ICC) of 0.05 to adjust for clustering, an anticipated 10% nonresponse rate, and a 10% loss to follow-up, the calculation yielded a minimum required sample of 2610 high-COPD-risk participants with multimorbid mental health symptoms. To date, 3807 participants have been enrolled, exceeding the initial target and allowing for more robust statistical analyses of the outcomes.

### Power Calculations

In our power calculation, we calculated the minimum detectable differences for the primary outcomes (WEMWBS, PHQ-9, and GAD-7). The ICC estimate was informed by previous literature, which suggests a likely ICC range of 0.03-0.05 for these outcomes [40,41]; we therefore selected 0.05 as a reasonable and conservative estimate. Using this ICC value, together with a 2-sided α of 0.05 and 80% power, the study is able to detect a standardized effect size of Cohen *d*=0.425. This corresponds to a small effect size and to the following raw score differences (Table 4): 4.497 for WEMWBS, 1.606 for PHQ-9, and 2.293 for GAD-7. These calculations indicate that the study is adequately powered to detect small but meaningful differences in these outcomes. Moreover, based on previous research reporting an effect size of 5.95 on the WEMWBS, we consider the study to be well powered to detect clinically relevant changes in these measures [39].

**Table 4:** Minimum detectable differences in WEMWBS^a^, PHQ-9^b^, and GAD-7^c^.

N_actual	Intraclass correlation coefficient	Cohen *d*	WEMWBS	PHQ-9	GAD-7
3807	0.050	0.425	4.497	1.606	2.293

^a^WEMWBS: Warwick-Edinburgh Mental Well-Being Scale.

^b^PHQ-9: 9-item Patient Health Questionnaire.

^c^GAD-7: 7-item Generalized Anxiety Disorder.

### Blindness

In this study, we employed an open-label design, in which both participants and health care providers were aware of group assignments. However, to reduce potential bias, several measures were implemented. Although participants and health care providers knew the intervention group allocation, the principal investigator remained blinded to group assignments. Given the trial design, principal investigator blinding represents the best possible approach and helps ensure impartiality in monitoring and data analysis.

Despite these efforts, complete blinding of both physicians and participants was not feasible due to practical constraints. Physicians, as key members responsible for administering the intervention, must be aware of group assignments to provide appropriate care. Additionally, participants are likely to be aware of their group assignment, particularly in psychological or behavioral interventions. Traditional clinical trials often highlight the risk of bias associated with a lack of blinding, especially for self-reported outcomes. However, open-label trials are frequently used in population medicine and real-world research settings, where blinding participants and health care providers is often impractical.

Given that our study includes 3 primary outcomes and 34 secondary outcomes, of which 8 are based on validated self-reported scales and 8 are based on objective assessments, we made efforts to include as many objective measures as possible to reduce bias. This approach enhances the objectivity of the data and minimizes reliance on self-reported outcomes.

Additionally, recognizing that social desirability bias may affect self-reported outcomes, we plan to conduct qualitative interviews as part of the follow-up assessments. These interviews will provide insights into the potential impact of social desirability bias on participants’ responses and help clarify how such biases may have influenced the results. By doing so, we aim to ensure a more comprehensive and balanced interpretation of the findings, while acknowledging the potential influence of bias on self-reported data.

### Assignment of the Intervention

This stratified cRCT assigned participants at the township level to either a multicomponent intervention or a control arm, with randomization stratified by geographic region to balance community characteristics. Using a computer-generated algorithm developed by an independent statistician not involved in recruitment or intervention delivery, 13 townships were allocated to each study arm. Given the open-label design, blinding was not feasible, and both participants and providers were aware of group assignments. Control group participants received no additional interventions beyond ongoing usual care throughout the trial period.

Adherence reinforcement strategies are embedded across all follow-up phases. A structured 3-month telephone consultation reinforces participation. In-person 6- and 12-month visits provide individualized feedback on key health indicators (eg, spirometry results, mental health scores), benchmarking progress against baseline values. Adherence is quantitatively tracked using validated questionnaires at each interval to assess protocol compliance (eg, app engagement frequency). Automated reminders and tailored counseling iteratively address adherence gaps. Each follow-up thus serves as an opportunity to enhance participant motivation, resolve barriers, and refine engagement strategies.

### Data Collection Plan

Data are collected across multiple domains to comprehensively assess the effects of the multicomponent intervention (see Table 1). At baseline, all participants completed a structured questionnaire that includes measures of mental health status (PHQ-9 for depression, GAD-7 for anxiety, and the Warwick-Edinburgh Mental Well-Being Scale for overall mental health), sociodemographic characteristics, occupational and environmental exposures, health-related behaviors, chronic disease history, and respiratory symptoms. Physiological and biochemical indicators include measurements of height, weight, BMI, waist circumference, blood pressure, heart rate, capillary blood glucose, and oxygen saturation.

Lung function is assessed using portable spirometry (FEV_1_, FVC, and FEV_1_/FVC) at multiple time points. In the high-COPD-risk population, prebronchodilator spirometry was performed at baseline and is repeated at months 6 (patients with COPD) and 12 to monitor changes in lung function. Among participants with diagnosed COPD with mental health symptoms, postbronchodilator spirometry was conducted at baseline and is repeated at month 12 to assess reversibility and disease progression.

Subsequent follow-up collections are scheduled at 3 months (via telephone), 6 months (in-person), and 12 months (in-person) for the intervention group and only at 12 months for the control group (in-person). These include assessments of mental health (PHQ-9, GAD-7, and WEMWBS), health-related quality of life (EQ-5D-5L), and Work Productivity and Activity Impairment-General Health. Complementary assessments encompass treatment adherence, control of COPD symptoms, frequency of exacerbations, physical and biochemical parameters (BMI, blood pressure, and blood glucose), and lung function, as previously specified. Lifestyle practices, including physical activity levels, alcohol intake, and dietary patterns (sugar consumption, salted vegetable intake, and fresh vegetable consumption), are additionally documented using participant self-reported questionnaires. Data on health care resource utilization, such as outpatient visits, hospital admissions, and medication use, are also systematically recorded.

All data collection is conducted by trained research personnel in accordance with standardized operational protocols. Collected data are entered into a secure cloud-based electronic data capture (EDC) platform equipped with automated validation checks and quality assurance procedures. These safeguards are designed to ensure data accuracy and minimize transcription errors.

### Data Management

This research employs a cloud-based EDC platform to facilitate immediate data entry, automated validation checks, and secure data storage. Trained field researchers collect information using tablet-based digital questionnaires, with responses synchronized to a central repository in real time. The EDC system incorporates built-in safeguards, including range validations, logical consistency checks, and alerts for incomplete entries, to reduce data entry errors. Any anomalies identified by these mechanisms are promptly reviewed by the study’s data management team.

Data collection instruments follow standardized, precoded formats to streamline demographic, clinical, and behavioral assessments. Biometric measurements (eg, lung function, blood pressure, BMI, and blood glucose) are entered directly into the system. Pulmonary function data from portable spirometry devices are transmitted to the EDC platform, where they undergo automated quality control procedures to verify data validity.

To protect participant confidentiality, all data are deidentified using numeric coding systems and stored in a password-protected database reserved exclusively for analytical purposes by the research team. Access to this database is strictly limited to a small group of authorized personnel, including the principal investigator, co-investigators, biostatisticians, and data analysts.

### Statistical Analysis

Primary analyses will estimate the effect of the intervention on the 3 primary mental health outcomes (WEMWBS, PHQ-9, and GAD-7 scores at 12 months) using generalized linear mixed models under the intention-to-treat principle. Before model fitting, baseline characteristics of participants in the intervention and control arms, including WEMWBS, PHQ-9, and GAD-7 scores, will be summarized to assess postrandomization balance and identify any substantial differences that may warrant additional covariate adjustment. Models will include a fixed effect for trial arm, visit, and trial arm-by-visit interaction; random intercepts for township (cluster) and participant to account for clustering and repeated measurements; and adjustment for the baseline value of the corresponding outcome and a set of prespecified baseline covariates. These covariates will include age, sex, smoking status, and other relevant demographic and health variables that may influence the outcomes. Continuous secondary outcomes will be analyzed using analogous linear mixed models, and binary outcomes using logistic mixed models with the same random-effects structure and covariate set.

To control for multiple comparisons across the 3 primary outcomes, we will apply the Bonferroni correction, dividing the significance threshold by 3 to reduce the family-wise error rate. For secondary outcomes, we will apply the Benjamini-Hochberg procedure at a 5% false discovery rate. False discovery rate control provides a principled and widely accepted way to limit the overall proportion of false positives, while preserving greater statistical power compared with more stringent approaches such as the Bonferroni correction.

Although the primary analysis focuses on the overall intervention package, we will also conduct additional secondary analyses using the Regression Discontinuity Design. This approach was used to explore the distributional effects of the intervention, with a specific focus on the WEMWBS outcome. We applied a threshold of 45 for WEMWBS scores, dividing participants into those just above and just below this cut-off. The Regression Discontinuity Design methodology allows us to estimate the local average treatment effect at this threshold, helping us assess the effects of the intervention on individuals near the cut-off and providing a more precise estimate of the intervention’s impact. By analyzing participants around this threshold, we evaluate the intervention’s effect in a more localized and targeted manner.

### Monitoring

An independent Data and Safety Monitoring Board (DSMB) was appointed to ensure scientific rigor and impartial oversight of the trial’s safety and progress. The DSMB convened 3 meetings in which baseline and follow-up descriptive analyses for the intervention arm were evaluated for process monitoring, as well as a formal interim analysis in February 2026. At this meeting, the research team reviewed the conduct of the trial, including adherence to intervention components, logistical issues, and the completeness and quality of the collected data. Our biostatisticians also presented emerging effect estimates for the intervention and control arms to the steering committee. Stopping rules were established regarding safety and study completion:

- If deaths or severe adverse effects were reported, these would be immediately conveyed to the DSMB. The DSMB would determine whether the deaths or adverse effects were related to the intervention and advise whether the study should be stopped immediately.
- In February 2026, during the planned interim analysis, the trial was approaching completion, as field workers confirmed that the majority of eligible participants had been contacted. At this stage, the results were assessed to determine the study’s conclusion based on the following:
  Null findings, suggesting that the intervention had no detectable effect and that the study should be stopped.
  Statistically significant findings, indicating that the study had achieved its primary objectives and should be stopped.
  Emerging but statistically insignificant effects, suggesting that the study might require further observation, if applicable. However, the study would be terminated if field workers confirmed that all eligible participants had been contacted and no additional individuals remained for follow-up assessments.

### Ethics and Dissemination

#### Research Ethics Approval

This study is registered on ClinicalTrials.gov under the identifier NCT06458218, with registration completed on June 9, 2024. It was initially approved by the Ethics Committee of Peking Union Medical College (approval number CAMS&PUMC-IEC-2024-043). A continuing ethics review was completed in June 2025, and updated approval was granted under the number CAMS&PUMC-IEC-2025-064. The study is conducted in accordance with the Declaration of Helsinki and good clinical practice. Participation in this study is entirely voluntary. The study was fully explained to participants, who were required to provide written informed consent before enrollment. Participants were required to declare that they freely and voluntarily agreed to participate in the study and to complete all required questionnaires. Participants were informed that they may withdraw from the study at any time without any repercussions. They may withdraw without providing a reason, and their data will be excluded upon request.

#### Plans for Communicating Important Protocol Amendments to Relevant Parties

The Ethics Committee of Peking Union Medical College was approached and informed of all protocol revisions requiring its review and approval. After securing Ethics Committee clearance but before implementation, all study personnel received detailed briefings on the amended protocol.

In this trial, an unavoidable amendment was made to the secondary outcome measures. Although outcome modifications are generally discouraged, the change was necessitated by a nonnegotiable regulatory restriction. Specifically, we initially planned to measure health care utilization (ie, outpatient visits, inpatient visits, and medical expenditure) via electronic health records from medical institutions in Xishui County. However, due to unexpected government regulations, we were unable to reach an agreement to obtain these data. This amendment was approved by the Ethics Committee, and all research personnel were notified before implementation to ensure consistency in data collection and analysis.

#### Consent and Withdrawal

Our study adheres to the ethical principles outlined in the Declaration of Helsinki and the Belmont Report, ensuring that all participants are treated with respect for autonomy, beneficence, and justice. Before enrollment, all eligible participants received detailed written study information and an informed consent form explaining the study’s purpose, procedures, potential risks, and benefits. Trained study investigators provided verbal explanations, in the presence of a witness when required, ensuring that participants fully understood their rights before providing consent. Participants were given sufficient time to consider their decision and had the opportunity to ask questions before signing the consent form. For illiterate participants, a thumbprint and a witness signature were obtained as a formal record of consent.

Participants provided written consent for the collection and analysis of biological samples (if applicable) and other relevant study data. They were informed that participation in the study is voluntary and that they have the right to withdraw consent at any time without facing any negative consequences related to health care access or other personal matters. In the event of withdrawal, participants may request the deletion of their collected data and the destruction of any stored biological samples, which will be carried out in accordance with ethical guidelines.

Throughout the study, participants will be informed about any abnormal findings identified from data collection. When relevant, they will be referred to appropriate health care facilities for further evaluation and treatment. Study participation will not interfere with any ongoing medical care, and participants will continue receiving their routine health care services. The intervention is designed to be low risk, ensuring that no harm is caused to participants. If any damage occurs to intervention components (eg, monitoring devices or other study-related materials), repairs or replacements will be provided as necessary.

Eligible households were fully informed about the potential risks and benefits of participation. No direct financial compensation was provided for participation, but material costs for intervention-related maintenance (if applicable) may be covered. Participants have the freedom to refuse or discontinue participation at any point without justification. The study team will track and document reasons for withdrawal and report findings in subsequent publications. No invasive procedures will be conducted beyond standard diagnostic assessments, and no additional interventions will be performed outside the scope of this study.

#### Confidentiality

To ensure the privacy and confidentiality of participants, all data collected in this study are entered into the EDC system via tablets and securely shared through the EDC platform. All data are encrypted and stored on a secure server at the Information Center of the Chinese Academy of Medical Sciences, in strict compliance with national data protection regulations. Personal identifiers are stored separately from health data, using a coded numerical system for analysis. Only authorized research personnel have access to identifiable data, and all information is stored in a password-protected database with restricted access. Before any publication or public dissemination, all participant information will be fully anonymized. The study strictly adheres to ethical guidelines, ensuring that participants’ rights and confidentiality are protected throughout the research process.

## Results

Data collection for this trial began in June 2024 and is planned to end by March 2026. Baseline assessments, 3-month follow-ups, and 6-month follow-ups have been completed, and 12-month follow-up assessments are currently underway as scheduled. Primary and secondary outcome analyses are expected to be completed in 2026, with the main trial results anticipated to be disseminated in 2027.

## Discussion

### Overview

This protocol reports a large cluster randomized trial designed to evaluate whether a multicomponent, population medicine–oriented intervention can improve both mental health and COPD-related outcomes among adults at high risk for chronic respiratory disease in a resource-limited rural setting. We anticipate that integrating digital psychological support, community-based screening and follow-up, and a pay-for-population incentive mechanism into routine primary care will lead to meaningful improvements in depression, anxiety, and overall mental well-being, while simultaneously strengthening COPD case-finding, diagnosis, treatment uptake, and disease control. These hypotheses provide the conceptual basis for the discussion below and frame the potential contributions of the POPMIX-MH trial.

This trial makes several contributions that distinguish it from previous COPD- or mental health–related cRCTs. First, it focuses on a high-risk but understudied population in rural China—namely, individuals at high risk for COPD who also experience psychological distress—and evaluates an integrated package that jointly targets mental and respiratory health. Second, the intervention operationalizes population-based chronic care by combining community screening, digital CBT, chronic disease management, and a pay-for-population incentive model within routine primary care. Third, unlike most COPD trials that focus primarily on physiological indicators, this study positions mental well-being and common mental disorder symptoms as primary outcomes, thereby broadening the goals of chronic disease management. Together, these features provide rare real-world evidence on a scalable, multimorbidity-oriented intervention in a resource-limited setting.

### Conceptual Contribution and Trial Rationale

Guided by the principles of population medicine, this study is the first to operationalize the modular, cost-effective population health intervention strategies proposed by the Lancet Commission on Investing in Health in a real-world setting—Xishui County, Guizhou Province, China [4]. Targeting individuals with mental health symptoms at high risk for COPD, the intervention integrates multiple components, including digital psychological support, health education, lung function screening, and structured follow-up. This design moves beyond the conventional disease-specific chronic care model and embodies the population medicine philosophy of addressing population-level health rather than individual illness alone [3]. As a designated national pilot zone for integrated primary health reform, Xishui County offers an ideal setting for testing the feasibility and policy relevance of such innovative strategies in a resource-limited rural context, marking the first real-world implementation of the Lancet Commission on Investing in Health framework.

### Strengths and Limitations

Unlike most chronic disease intervention studies that primarily focus on physiological outcomes, such as lung function or exacerbation rates, the POPMIX-MH trial places mental health at the core of its outcome framework [42,43]. Using standardized instruments such as the PHQ-9, GAD-7, and WEMWBS, the study comprehensively assesses changes in depression, anxiety, and overall mental well-being. This approach directly responds to the high prevalence of underrecognized psychological distress among individuals at high risk for COPD, challenging the long-standing emphasis on organ-specific metrics in chronic disease care [44,45]. By combining digital CBT interventions (via EmoEase) with personalized health education, the program not only improves psychological outcomes but also enhances participants’ understanding of their condition and self-management capabilities—factors that may, in turn, contribute to better physical health. Conceptually, this expands the definition of health in chronic care; operationally, it provides a scalable model for embedding mental health support into primary care systems.

While mental health outcomes constitute the primary end points of this study, a comprehensive set of secondary outcomes, such as COPD, asthma, hypertension, and diabetes care cascade performance; health care resource utilization; knowledge; high-risk behaviors and lifestyles; and socioeconomic profiles, has been included to capture the broader impact of the intervention across multiple dimensions of population health, including physical, mental, social, and environmental well-being [46].

The multicomponent intervention strategies employed in this study have all been recognized by the Lancet Commission on Investing in Health as cost-effective and high-priority [4]. In addition to digital health interventions, the package includes population-based screening, intensive counseling and follow-up, and a pay-for-population incentive mechanism for community health workers. Among these, population-based COPD screening, although proven to be cost-effective through the simulation model, has rarely been implemented at scale in real-world settings [47]. One of the key objectives of this study is to pilot this screening approach in rural regions such as Xishui County, enabling early identification and timely referral of suspected patients to higher-level hospitals for formal diagnosis and standardized treatment. Intensive counseling and regular follow-up are also core components of this study and are designed to ensure adherence to and the sustainability of the intervention. With ongoing psychological support, lifestyle guidance, and disease monitoring, participants are better equipped to maintain positive behavioral changes throughout the intervention period. These activities also represent fundamental responsibilities of community health workers and primary care physicians, as the long-term management of chronic and multimorbid conditions relies heavily on patients’ compliance with medical advice and consistent engagement with follow-up care. To motivate active service delivery and transform traditional care models, this study implemented a pay-for-population incentive mechanism. Under this system, performance-based payments are tied to key health indicators, such as screening rates, diagnosis rates, treatment uptake, and disease control. This model encourages a shift from conventional “clinic-based passive care” to “proactive community-based service,” prompting health care workers to go beyond the clinic to conduct screening and health promotion directly within the population.

### Policy Implications

Our trial has strong policy implications. First, this study aims to systematically assess the feasibility and contextual adaptability of a multicomponent, population-based intervention within primary and community health systems in China and beyond. Unlike traditional single-disease, facility-centered models of chronic care, our approach emphasizes real-world integration of psychological support, behavior modification, disease screening, and workforce incentives into existing community health infrastructure. Experiences from this trial may offer practical guidance for scaling up community-based population health strategies in resource-limited and high-burden contexts similar to Xishui County, where the study is being conducted. Second, this study seeks to address a historically underappreciated area in chronic disease management: the widespread but unrecognized mental health burden among high-COPD-risk populations. By incorporating tools such as the PHQ-9, GAD-7, and WEMWBS into screening and follow-up workflows, we aim to support comprehensive assessment of participants’ psychological status and apply scalable digital CBT-based interventions. This represents not only a technical innovation but also a conceptual shift, positioning mental health as a core component of chronic care, equal in priority to physiological outcomes.

Most importantly, we hope this study will catalyze a paradigm shift in the role of primary and community health workers. In conventional health systems, frontline providers often operate passively, waiting for patients to present at clinics. This trial introduces mechanisms such as pay-for-population, performance-linked process metrics, and home-based outreach to actively engage providers in population screening, health promotion, and continuous care. This transformation—from a patient-centered, reactive model to a proactive, population-centered approach—is at the heart of population medicine. Although this trial is conducted in a single rural county in southwest China with a high burden of COPD risk and common mental health problems, our first priority is to establish the effectiveness of the intervention within Xishui itself through a science-first, real-world evaluation. Given economic, cultural, and health-system differences across regions, adaptations will likely be required for implementation elsewhere. Nevertheless, we believe that the population-centered approach tested in this study has the potential to reshape public health governance not only in similar resource-limited primary care settings in China but also in other low- and middle-income countries striving for equitable, sustainable health system reform.

### Dissemination

Upon completion of the trial, participant-related information and results will be provided to participants themselves or their families. The trial has gained public attention since it was first introduced in the Lancet Commission on Investing in Health. We will disseminate the results of the trial in international peer-reviewed journals and at other renowned international academic conferences. Authorship will be determined according to the International Committee of Medical Journal Editors (ICMJE) criteria. If any professional medical writers are used, their involvement will be transparently disclosed in the publication.

### Conclusion

This trial represents a pioneering effort to integrate population medicine principles into the management of common mental health conditions in resource-limited rural settings. By combining community-based screening, digital CBT interventions, and team-based care supported by performance-linked incentives, the study aims to improve early detection, enhance access to effective treatment, and strengthen continuity of care. The findings are expected to provide robust evidence for scalable, cost-effective mental health service models and to inform national and international policy development, promoting equitable and sustainable improvements in population mental health.

## Appendix

Multimedia Appendix 1 Standard Protocol Items: Recommendations for Interventional Trials (SPIRIT) 2025 checklist.

Multimedia Appendix 2 The pay-for-population incentive mechanism.

Multimedia Appendix 3 Secondary outcomes and trial timeline.

Multimedia Appendix 4 Full size versions of Figures 1–3.

## Abbreviations

CBT: cognitive behavioral therapy
COPD: chronic obstructive pulmonary disease
COPD-SQ: COPD Screening Questionnaire
cRCT: cluster randomized controlled trial
DSMB: Data and Safety Monitoring Board
EDC: electronic data capture
FEV1: forced expiratory volume in 1 second
FVC: forced vital capacity
GAD-7: 7-item Generalized Anxiety Disorder
ICC: intraclass correlation coefficient
NCD: noncommunicable disease
PHQ-9: 9-item Patient Health Questionnaire
POPMIX: Population Medicine Multimorbidity Intervention in Xishui County
SPIRIT: Standard Protocol Items: Recommendations for Interventional Trials
WEMWBS: Warwick-Edinburgh Mental Well-Being Scale

## Disclaimer

The authors declare the use of generative artificial intelligence in the research and writing process. According to the GAIDeT (Guideline for Reporting Evidence-based Practice Educational Interventions and Teaching) standards (2025), the following tasks were delegated to generative artificial intelligence tools under full human supervision: proofreading and editing, adapting and adjusting emotional tone, reformatting, and quality assessment. The generative artificial intelligence tools used were ChatGPT 5.1 (OpenAI) and DeepSeek (High-Flyer). Responsibility for the final manuscript lies entirely with the authors. Generative artificial intelligence tools are not listed as authors and do not bear responsibility for the final outcomes.

## Data Availability

The datasets used or analyzed in this study are available from the principal investigator (CW) on reasonable request.
