# Supplementary material for: Impact of the Population Medicine Multimorbidity Intervention in Xishui County (POPMIX) on People at High Risk for Chronic Obstructive Pulmonary Disease Who Experience Mental Health Symptoms: Protocol for the POPMIX-MH Cluster Randomized Controlled Trial
Source: JMIR Res Protoc. 2026 Mar 6;15:e85853. doi: 10.2196/85853 (PMC13005060; doi:10.2196/85853)
Supplement: Multimedia Appendix 2 [file resprot_v15i1e85853_app2.docx]

**Design and Implementation of the "Pay-for-Population" Incentive Mechanism**

**in the POPMIX Study**

The "pay-for-population" incentive mechanism in this study has received significant attention from reviewers, particularly concerning the structure of rewards, funding, motivation intensity, implementation methods, and the specific roles of healthcare workers. To enhance the transparency and replicability of our research and directly address these important questions, this supplementary material outlines the mechanism's overall design, operation process, and detailed structure. We will also clarify the funding allocation, along with the mechanism's suitability and potential risks when implemented in resource-constrained settings.

1. **Overall Design Logic of the Pay-for-Population Mechanism**

The POPMIX trial utilizes an incentive model that intentionally diverges from the traditional "Pay-for-Performance" (P4P) approach, which typically focuses on clinical outcomes. Instead, our design is based on the "Pay-for-Population" model, directly linking incentives to population coverage of care. Traditional P4P often ties payments to clinical parameters, such as blood pressure, blood glucose control, or emergency exacerbation rates; these metrics are closely associated with individual patient outcomes. This established approach, however, can inadvertently promote "selective care" or the "management of easier-to-treat populations," often resulting in suboptimal motivational responses .[1-3] In contrast, the motivation mechanism in this study is entirely unrelated to clinical outcomes like these, and is instead associated with the intervention coverage related to COPD care at the population level.

In the POPMIX trial, the performance indicators at the county level are structured around the COPD care cascade, covering four sequential phases: screening, diagnosis, treatment, and control management. These indicators specifically measure management coverage across high-risk screening, diagnosis rates, treatment rates, and control rates. One million yuan has been specifically allocated for the incentive design and weighted according to the relative importance of these phases: 50% for screening, 25% for diagnosis, 15% for treatment, and 10% for control. The performance indicators for all four phases operate within a consistent, progressive framework, yet each uses a distinct denominator. For instance, the screening indicator uses the resident population aged 35 and older in the 13 intervention townships as the denominator, reflecting the coverage of the high-risk population. Progression to the diagnosis phase uses high-risk residents those with a COPD-SQ score of 16 or above in the 13 townships as the denominator, thereby reflecting the confirmation rate among high-risk individuals. The treatment phase employs confirmed COPD patients in the 13 intervention townships as the denominator to assess the proportion receiving standardized inhaled treatment. Similarly, the control phase also uses confirmed patients as the denominator, assessing management coverage for those who did not experience an acute exacerbation in the past six months (see Table 1).

It is crucial to emphasize that these indicators measure whether care is provided, not whether the care is “effective”. In other words, this study prioritizes ensuring that more residents actually receive basic and necessary chronic disease management, without holding healthcare providers responsible for the physiological disease outcomes of individual patients. This design aspect makes our incentive system inherently fairer for grassroots healthcare workers and uniquely suited for resource-limited counties that face high levels of complex disease and significant care burdens.

**Table 1: Performance Indicators for the Four Stages of the COPD Care Cascade and the Weights in Pay-for-Performance Incentives**

| **Indicator** | **Objective** | **Standard** | **Indicator Weight** |
| --- | --- | --- | --- |
| Screening | Number of individuals aged ≥35 years who completed the initial screening questionnaire (COPD-SQ) in the selected 13 townships / Total number of individuals aged ≥35 years in the 13 townships (%) | In the budget calculation, this project allocates RMB 1,000,000 as incentives for the County Hospital (Respiratory Department), County Center for Disease Control and Prevention(CDC), and the 13 intervention township health centers. Among them, RMB 500,000 is allocated for screening, RMB 250,000 for diagnosis, RMB 150,000 for treatment, and RMB 100,000 for disease control. | 50% |
| Diagnosis | Number of individuals in the 13 townships who completed spirometry testing / Total number of high-risk individuals who had positive initial screening results in the 13 townships (COPD-SQ >16) (%) |  | 25% |
| Treatment | Number of diagnosed COPD patients in the 13 townships who used inhaled medications / Total number of diagnosed COPD patients in the 13 townships (%) |  | 15% |
| Control | Number of diagnosed COPD patients in the 13 townships who did not experience acute exacerbations in the past 6 months / Total number of diagnosed COPD patients in the 13 townships (%) |  | 10% |

While the County Hospital’s Respiratory Department, the County CDC, and the 13 Township Health Centers are all encompassed within a unified performance framework, we differentiate the incentive calculation criteria. This approach ensures that the core assessment content remains precisely aligned with the respective responsibilities of county-level institutions versus township health centers.

***(1) Incentive Calculation Method for Township Health Centers***

Incentives for Township Health Centers are determined by the population under their care including residents aged 35 and above, high-risk individuals, and already diagnosed patients with the final incentive amount calculated based on their completion rate across the four key indicators at the township level. This specific method highlights the primary responsibility of the Township Health Centers in community mobilization, high-risk identification, and follow-up management, alongside measuring their overall coverage efficiency.

***(2) Incentive Calculation Method for County Hospital's Respiratory Department and County Disease Control Center***

County-level institutions receive incentives based on the overall completion rate of county-wide indicators, reflecting their critical roles in training, quality control, data management, and county-wide coordination. Specifically, the County Hospital’s Respiratory Department handles diagnosis and professional support, while the County CDC is responsible for regional data aggregation and quality assessment. By utilizing county-wide data, we ensure that assessment biases resulting from inherent differences between townships are effectively avoided.

This hierarchical assessment system drives coordinated progress toward shared population health goals between county-level institutions and township health centers. By ensuring that each assumes responsibilities aligned with their functions, we realize organizational collaboration under the framework of population medicine.

The coverage-based incentive model offers a significant advantage: it possesses the capacity to proactively expand the reach of healthcare resources. Incentives linked to the scope of care coverage, as opposed to individual outcomes, inherently motivate healthcare workers. This motivation encourages them to actively identify and engage community members who are undiagnosed, untreated, or not receiving standardized care. Furthermore, the marginal cost associated with community screening and follow-up is relatively low. Once operational workflows are established, 'screening one more person' does not result in a significant increase in workload , but rather facilitates the identification of more individuals with unmet health needs. Consequently, the population receiving care under the POPMIX model often extends beyond the strict boundaries of the research sample, incorporating a broader demographic previously excluded from the healthcare system.

1. **The Mechanisms of Incentive Effects on Healthcare Workers: Dual Pathways of Extrinsic and Intrinsic Motivation**

The POPMIX incentive system does not rely solely on financial rewards (extrinsic motivation) to drive behavioral change. Instead, it is designed to integrate these financial rewards with intrinsic motivation, thereby achieving more sustainable behavioral changes in resource-constrained environments.

1. ***Extrinsic Motivation***

At the level of extrinsic motivation, incentive funds tied to coverage rates furnish grassroots institutions with additional performance resources. For township health centers, the incentive amount is directly proportional to the population size and the completion rate within their jurisdiction. This structure transmits a clear economic signal at the institutional decision-making level, and subsequently, through internal performance distribution mechanisms, it enhances the motivation of frontline healthcare workers to actively participate in community screening, follow-up, and health education activities. Furthermore, the incentives provided to the County Hospital’s Respiratory Department constitute a certain proportion of its annual departmental income, helping the specialty extend its focus from routine outpatient and inpatient care to chronic disease management and capacity building at the county level.

1. ***Intrinsic Motivation***

At the level of intrinsic motivation, the program continuously enhances healthcare workers' professional capabilities in disease management through systematic training, regular quality feedback, and data visualization. The resulting skill improvements foster a sense of competence; concurrently, the autonomy granted to township health centers in organizing their services strengthens their sense of work autonomy. Furthermore, the value orientation inherent in the population medicine framework (focusing on "providing more residents with the health services they deserve") significantly reinforces their professional mission and achievement.

To gain a deeper understanding of this intrinsic motivation, the study plans to conduct in-depth interviews. These interviews will specifically explore how staff members experience and exercise their professional autonomy, sense of competence, and sense of purpose in their daily work, and how these factors stimulate their active participation and behavioral changes in chronic disease management. This approach will clarify the specific pathways through which intrinsic motivation functions among grassroots healthcare workers, thereby revealing how the entire incentive mechanism impacts the sustainability of long-term behavioral changes.

The strategic combination of extrinsic and intrinsic incentives provides healthcare workers with both the direct drive of "receiving additional compensation" and the crucial value alignment of "doing the right thing" which promotes the formation of relatively stable behavioral changes.

1. **Impact on Routine Medical Care and Control of Resource Substitution Risks**

In resource-limited county-level environments, introducing new incentive mechanisms and interventions always carries the risk of resource substitution, which can potentially reduce care access for non-participants or weaken other public health services. To proactively mitigate this concern, we engaged in full consultation with the County Health Bureau during the POPMIX design phase. This ensured that the project would not require increased staffing levels or changes to the existing outpatient duty system. Furthermore, we actively integrated activities such as screening, follow-up, and health education into the existing workflow wherever feasible. The intervention was thus framed as a structured strengthening of daily responsibilities, rather than the addition of a new, separate project. At the same time, all training and feedback sessions were deliberately scheduled during regular working hours, preventing the long-term diversion of healthcare workers from their core clinical tasks.

The population-based payment incentive mechanism is the central tool for embedding the principles of population medicine into the county-level healthcare system within the POPMIX design. The core strategy is linking incentives to the coverage rate of key population segments (including high-risk screening, pulmonary function testing, standardized treatment, and follow-up management), rather than tying rewards to the short-term clinical outcomes of individual patients. This mechanism motivates grassroots healthcare institutions to expand their focus from individual patients to the broader target population of their jurisdiction, while simultaneously fulfilling their existing public health and chronic disease management responsibilities.

Consequently, performance assessment shifts: healthcare workers no longer evaluate success primarily based on "whether a specific patient has been diagnosed and treated," but instead assess system-wide coverage by asking, "what proportion of the population in the jurisdiction who should receive screening, diagnosis, and standardized management have actually received these services?". This population coverage-based incentive arrangement naturally integrates individual clinical activities into a continuous health management chain aimed at the entire population. This outcome effectively reflects the fundamental approach of population medicine, which combines clinical medicine with public health practices.

1. Eijkenaar, F., *Pay for performance in health care: an international overview of initiatives.* Medical Care Research and Review, 2012. **69**(3): p. 251-276.

2. Mathes, T., et al., *Pay for performance for hospitals.* Cochrane Database of Systematic Reviews, 2019(7).

3. Eijkenaar, F., *Key issues in the design of pay for performance programs.* The European Journal of Health Economics, 2013. **14**(1): p. 117-131.
