# Supplementary figures and images for: Impact of the Population Medicine Multimorbidity Intervention in Xishui County (POPMIX) on People at High Risk for Chronic Obstructive Pulmonary Disease Who Experience Mental Health Symptoms: Protocol for the POPMIX-MH Cluster Randomized Controlled Trial
Source: JMIR Res Protoc. 2026 Mar 6;15:e85853. doi: 10.2196/85853 (PMC13005060; doi:10.2196/85853)

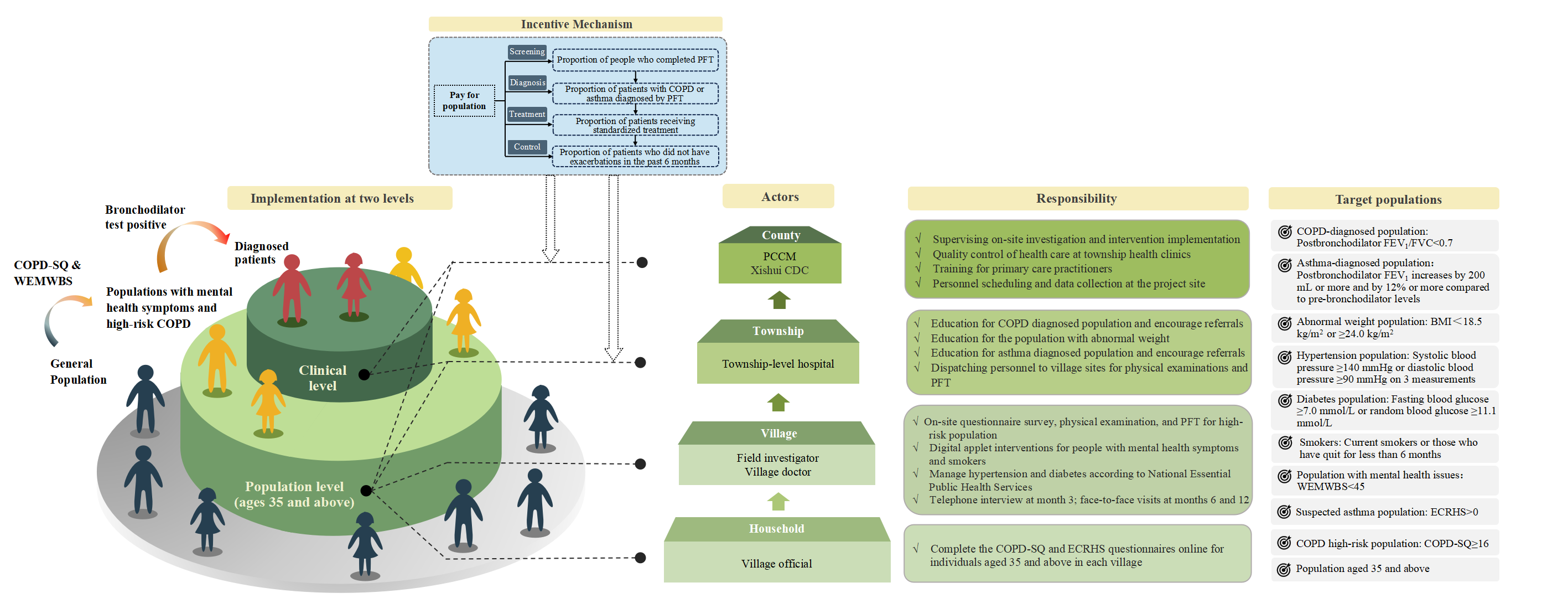

Supplement: Multimedia Appendix 4 [file resprot_v15i1e85853_app4.zip › MH_Fig1-3/Fig.3.tif]
